# Systemic Inflammation and Structural Retinal Damage on OCT in Acute Non-Arteritic Central Retinal Artery Occlusion—A Retrospective Study

**DOI:** 10.3390/ijms27072961

**Published:** 2026-03-24

**Authors:** Aurelia Mihaela Nica, Roxana Elena Ciuntu, Samer Andrei Nica, Cristina Gena Dascalu, Cosmin Victor Ganea, Mihaela Corlade-Andrei, Paula Cristina Morariu, Maria Mihaela Godun, Alexandru-Florinel Oancea, Dragos Traian Marcu, Camelia Margareta Bogdănici, Mariana Floria

**Affiliations:** 1 Grigore T. Popa University of Medicine and Pharmacy Iasi, 16 University Street, 700115 Iasi, Romania; nica_aurelia-mihaela@d.umfiasi.ro (A.M.N.); samer-andrei.nica@d.umfiasi.ro (S.A.N.); cristina.dascalu@umfiasi.ro (C.G.D.); cosmin-victor.ganea@umfiasi.ro (C.V.G.); mihaela.corlade2@umfiasi.ro (M.C.-A.); alexandru.oancea@umfiasi.ro (A.-F.O.); marius-traian-dragos.dm-marcu@umfiasi.ro (D.T.M.); floria.mariana@umfiasi.ro (M.F.); 2 Ophthalmology, 2nd Surgery Department, Saint Spiridon Emergency Hospital, 700111 Iasi, Romania; 3 Otorhinolaryngology, 2nd Surgery Department, Saint Spiridon Emergency Hospital, 700111 Iasi, Romania; 4 Emergency Medicine, 2nd Surgery Department, Saint Spiridon Emergency Hospital, 700111 Iasi, Romania; 5 Department of Internal Medicine, Saint Spiridon Emergency Hospital, 700111 Iasi, Romania

**Keywords:** central retinal artery occlusion, optical coherence tomography, systemic inflammation, inflammatory indices, retinal ischemia, neutrophil-to-lymphocyte ratio

## Abstract

Central retinal artery occlusion (CRAO) is an acute ophthalmic emergency causing irreversible vision loss due to retinal ischemia. Inflammation is increasingly recognized as a contributor to vascular occlusions, but the relationship between systemic inflammatory biomarkers and structural retinal damage in CRAO remains unclear. This retrospective study included 37 patients with non-arteritic CRAO presenting within 168 h of symptom onset. Optical coherence tomography (OCT) classified ischemic severity as mild, moderate, or severe based on inner retinal morphology. Systemic inflammatory indices, including neutrophil-to-lymphocyte ratio (NLR), systemic immune-inflammation index (SII), platelet-to-lymphocyte ratio (PLR), monocyte-to-lymphocyte ratio (MLR), and systemic inflammation response index (SIRI), were calculated from admission blood tests, and associations were analyzed using ordinal logistic regression. Increasing severity correlated with higher NLR and SII values (*p* < 0.05). Univariable analysis showed significant associations for NLR, SII, neutrophil count, white blood cell count, and central macular thickness (CMT). Multivariable regression identified NLR (OR = 8.398, *p* = 0.007) and CMT (OR = 1.052, *p* = 0.001) as independent predictors of greater ischemic damage. Other indices were not significant. These findings indicate that NLR is the primary systemic inflammatory predictor of OCT-defined severity in acute CRAO, with CMT providing complementary structural information.

## 1. Introduction

Central retinal artery occlusion (CRAO) represents an ophthalmic emergency and a rare but severe cause of sudden monocular vision loss, characterized by abrupt interruption of central retinal arterial perfusion with subsequent inner retinal ischemia [1]. Its annual incidence is estimated at about 1–2 per 100,000, increasing markedly with age [2].

Most cases are non-arteritic and are primarily attributed to thromboembolic mechanisms, with emboli arising from atherosclerotic disease of the ipsilateral carotid circulation or from cardiac sources [2,3]. The development of CRAO has been associated with a range of systemic vascular risk factors, such as smoking, hypertension, dyslipidemia, diabetes mellitus, elevated body mass index, coagulopathy, cardiac disease (notably atrial fibrillation) and a positive family history of vascular disease [2,3,4,5].

Systemic inflammation has been increasingly linked to retinal microvascular dysfunction, with inflammatory markers consistently associated with adverse changes in retinal vessel caliber, particularly venular dilation, reflecting impaired microcirculation [6]. Accordingly, indices derived from the complete blood count (CBC), such as the neutrophil to lymphocyte ratio (NLR), platelet to lymphocyte ratio (PLR), monocyte to lymphocyte ratio (MLR), systemic immune inflammation index (SII), and systemic inflammation response index (SIRI), have been proposed as accessible markers of inflammatory burden in retinal artery occlusion [7,8]. While several studies report elevated indices in central retinal artery occlusion, it is less clear how these markers relate to the extent of acute structural retinal damage. Recent evidence indicates that systemic inflammatory processes may also manifest in ocular fluids, with measurable changes observed in tear and serum crystallization patterns [9].

Optical coherence tomography (OCT) is a widely used noninvasive imaging technique that provides detailed structural information about the retina through high-resolution cross-sectional imaging, allowing for objective characterization of acute ischemic injury and severity stratification based on inner retinal changes [1,4,10]. Acute CRAO is characterized on OCT by inner retinal hyperreflectivity, thickening, and progressive loss of layer stratification [11,12,13]. Retinal thickening has been shown to evolve in a time-dependent manner after symptom onset, whereas later stages are typically marked by resolution of edema and subsequent inner retinal atrophy [14,15].

Therefore, the aim of this study was to evaluate the association between systemic inflammatory indices and optical coherence tomography-based severity in patients presenting with acute non-arteritic central retinal artery occlusion (NA-CRAO). We hypothesized that higher inflammatory indices are associated with more severe structural retinal involvement.

## 2. Results

### 2.1. Baseline Characteristics

Thirty-seven patients with acute NA-CRAO were included in the analysis. Based on OCT findings, ischemic severity was classified as mild in 7 patients (18.9%), moderate in 16 (43.2%), and severe in 14 (37.8%). Detailed baseline demographic and clinical characteristics are provided in Table 1.

The mean age was 65.9 ± 10.5 years, and most patients were male (64.9%). Baseline presentation parameters including laterality, intraocular pressure, best-corrected visual acuity (logMAR), and time from symptom onset were comparable across OCT severity groups (all *p* > 0.05). Conventional inflammatory markers, including C-reactive protein (CRP) and erythrocyte sedimentation rate (ESR), were within normal ranges across all groups and showed no statistically significant differences according to OCT-defined ischemic severity (CRP: *p* = 0.353; ESR: *p* = 0.309).

The distribution of cardiovascular risk factors was comparable across the different ischemic severity categories, although dyslipidemia showed a borderline association with OCT severity (*p* = 0.055). A cilioretinal artery was present in mild and moderate cases but absent in severe ischemia; however, the difference was not statistically significant (*p* = 0.068).

### 2.2. OCT Severity and Inflammatory Indices

Central macular thickness (CMT) increased significantly across OCT-based ischemic severity categories (*p* < 0.001), with the highest values observed in the severe group (Table 2).

Post hoc analyses showed higher CMT in severe ischemia compared with mild (*p* < 0.001) and moderate ischemia (*p* = 0.001).

Among the inflammatory markers analyzed, significant differences across severity groups were observed for neutrophil count (*p* = 0.013), NLR (*p* = 0.029), and SII (*p* = 0.018) (Table 3).

Post hoc comparisons indicated that both NLR and SII were significantly higher in the severe group compared to the mild group. WBC, monocytes, lymphocytes, platelets, MLR, PLR, and SIRI did not differ significantly across groups (all *p* > 0.05).

### 2.3. Univariable Regression Analysis

Ordinal logistic regression analysis was applied to assess the relationship between systemic inflammatory markers and OCT-based ischemic severity. In univariable analysis, CMT, NLR, SII, neutrophil count, and WBC were significantly associated with greater ischemic severity (*p* < 0.05). Among these, NLR exhibited the strongest effect size (OR = 2.659; 95% CI: 1.240–5.703; *p* = 0.012). This indicates that each one-unit increase in NLR was associated with a 2.659-fold higher odds of belonging to a more severe OCT ischemia category. In contrast, neither dyslipidemia nor the presence of a cilioretinal artery showed a significant association with ischemic severity (*p* > 0.05) (Table 4).

### 2.4. Multivariable Regression Analysis

Prior to multivariable modeling, correlations between candidate predictors were assessed using Spearman coefficients to minimize multicollinearity. Strong correlations were observed between NLR and neutrophil count (rho = 0.574, *p* < 0.001), as well as between NLR and SII (rho = 0.842, *p* < 0.001), whereas no significant correlation was found between NLR and CMT (rho = 0.153, *p* = 0.366). CMT showed a moderate correlation with WBC (rho = 0.355, *p* = 0.031). Based on these findings, the multivariable ordinal logistic regression model included NLR and CMT as independent predictors.

In this model, both variables remained independently associated with ischemic severity (Table 5).

## 3. Discussion

This study explored the association between systemic inflammatory markers and OCT-based structural severity in patients with acute NA-CRAO. To our knowledge, it is among the first studies to relate these indices to the extent of retinal tissue injury captured by OCT. We found that a higher systemic inflammatory burden, reflected by elevated NLR and SII values, was associated with more extensive ischemic damage. These findings suggest that certain inflammatory markers may reflect underlying pathophysiological processes contributing to retinal tissue injury, and thus hold potential value for early risk stratification. By integrating systemic inflammation markers with structural OCT findings, our results support the concept that systemic inflammation is not merely a consequence but potentially a contributor to ischemic burden in CRAO.

Our findings are consistent with a recent meta-analysis demonstrating significantly elevated NLR and PLR values in patients with retinal artery occlusion compared with controls. The pooled analysis of eight retrospective studies including 1444 participants reported a moderate to large effect size for NLR and a smaller but significant effect for PLR, despite substantial between-study heterogeneity [7].

Systemic inflammatory markers derived from the complete blood count have been widely investigated in CRAO, although their clinical utility varies substantially by marker and context. Among them, NLR has been most consistently reported to be significantly higher in CRAO patients versus controls [16,17,18,19]. As a composite marker reflecting neutrophilia and relative lymphopenia, NLR is considered a surrogate of innate immune activation and systemic stress, processes that are mechanistically relevant to acute arterial occlusion.

In our cohort, higher NLR values were observed with increasing ischemic severity, supporting a graded link between systemic inflammation and the extent of retinal damage assessed on structural imaging. This pattern suggests that NLR captures more than disease presence alone, instead reflecting an inflammatory response that scales with ischemic burden. This inflammatory milieu is thought to promote endothelial dysfunction, embolic propensity, and microvascular vulnerability, thereby predisposing to more extensive ischemic injury. Taken together, these findings point to a more prominent role of the neutrophil-related inflammatory response in the most severe CRAO presentations.

Central macular thickness represents a direct structural manifestation of retinal ischemic injury following abrupt perfusion failure [20,21,22,23]. The absence of correlation between NLR and CMT in our cohort, together with their additive effects in multivariable analysis, supports a complementary mechanistic framework in which systemic inflammatory burden and local retinal tissue response operate through distinct but convergent pathways. Notably, NLR emerged as the dominant systemic predictor, with each one-unit increase associated with an 8.398-fold higher odds of belonging to a more severe OCT-defined ischemia category, whereas CMT showed a smaller but stable independent association reflecting local tissue injury. Thus, while NLR may influence vascular vulnerability and microvascular injury, CMT provides tissue-level quantification of ischemic damage, and their combined assessment offers a more comprehensive stratification of disease severity in acute NA-CRAO.

Notably, Elbeyli et al. reported that red cell distribution width (RDW) showed greater discriminatory performance than NLR, and PLR and was the only independent predictor of CRAO in multivariable analysis, underscoring that different inflammatory markers may capture distinct aspects of the systemic response to retinal ischemia [17].

Platelet-to-lymphocyte ratio has been proposed as an index reflecting platelet activation alongside relative lymphopenia, linking inflammatory and prothrombotic pathways. However, evidence regarding PLR in RAO remains heterogeneous. While some studies reported higher PLR in affected patients than in controls, others found no meaningful differences [16,18]. Consistent with this variability, PLR was not associated with ischemic severity in our cohort, suggesting it may lack sensitivity in capturing the extent of retinal tissue damage.

Systemic immune-inflammation index has emerged as a versatile and accessible biomarker that reflects the interplay between systemic inflammation and immune status. Its clinical relevance has been demonstrated across multiple domains, including oncology, cardiovascular and cerebrovascular diseases, infectious conditions, and autoimmune disorders, where it correlates with disease severity, mortality risk, and prognosis [24,25,26,27]. In our cohort, higher SII values were associated with greater ischemic severity, reflecting the possible contribution of systemic inflammatory and thrombotic activity to the extent of retinal ischemic involvement. SII may thus serve as a useful tool for risk stratification and clinical monitoring in acute NA-CRAO.

In contrast, MLR and SIRI showed no significant association with ischemic severity, suggesting that not all systemic inflammatory indices equally reflect the mechanisms underlying retinal ischemic injury. In keeping with these observations, conventional inflammatory markers such as CRP and ESR were also not associated with OCT-defined ischemic severity and remained within normal ranges across all groups, further reinforcing the need to prioritize markers more closely linked to the underlying pathophysiology.

The present study provides novel insight into the clinical interpretation of systemic inflammatory indices in CRAO. While previous studies have primarily focused on the presence and elevation of inflammatory markers in CRAO, our study advances this perspective by demonstrating that these indices are not only elevated, but also proportionally associated with the degree of ischemic retinal injury, as assessed by OCT. This distinction is clinically meaningful, as it reframes inflammatory markers from simple indicators of disease presence to potential tools for quantifying tissue damage and stratifying acute ischemic severity.

Optical coherence tomography enables high-resolution, layer-specific visualization of ischemic injury in CRAO which directly reflects the extent of retinal damage [23,28,29,30,31]. Furthermore, recent studies recognize OCT as the primary modality for assessing ischemic injury severity, due to its capacity to detect early retinal edema, hyperreflectivity, and disorganization of inner retinal layers [32,33,34].

Mangla et al. [12] introduced a purely OCT-based morphological classification system for CRAO, addressing the heterogeneity and subjectivity of previous models, which relied on mixed clinical, angiographic, and imaging criteria and commonly categorized CRAO severity as incomplete, subtotal, or total. Their grading reflects a continuous progression of structural and pathological changes, closely aligned with capillary plexus involvement and the extent of ischemic retinal injury. Furthermore, the described changes follow a consistent temporal evolution, allowing for early detection and providing a practical framework for longitudinal assessment of disease severity and potential treatment response. For our study, we adopted this grading because it uses objective, routinely available features in acute care, providing a reproducible and quantifiable measure of severity.

In a recent retrospective study, Hu et al. [8] examined peripheral blood inflammatory indices in CRAO and related them to disease severity using a composite grading system that integrated fluorescein fundus angiography, funduscopic cherry-red spot, and OCT characteristics to classify cases as incomplete, subtotal, or total CRAO. NLR, SII, and MHR were higher in CRAO than in controls, but across severity grades, only MHR (driven by lower HDL-C) was associated with total CRAO, whereas NLR and SII did not significantly differ between subtypes.

While Hu et al. primarily characterize disease severity at the vascular and clinical level, our study focuses on the severity of tissue level ischemic damage, quantified on OCT. By defining severity exclusively through OCT-based morphological criteria, we sought to capture the downstream retinal consequences of ischemia, rather than upstream perfusion abnormalities. In this context, systemic inflammatory indices are interpreted in relation to structural retinal damage, providing a complementary perspective that does not depend on perfusion imaging. Thus, the two frameworks capture different dimensions of CRAO severity and are best viewed as complementary.

From a clinical perspective, integrating structural retinal injury on OCT with inflammatory indices adds contextual information that supports severity assessment and may inform subsequent systemic evaluation. This is feasible in routine acute care, since both OCT and complete blood count parameters are typically available without delaying workflows.

Our findings should be interpreted in light of several limitations. The retrospective design and modest sample size may limit statistical power and generalizability. In particular, the limited number of patients in the mild ischemic severity group may have affected the stability and precision of the regression estimates. This is reflected in the wide confidence intervals observed, especially for NLR. Therefore, these findings should be interpreted with caution, as they may be prone to variability. Nevertheless, the consistent direction of the observed associations suggests a potential signal despite the limited statistical power of the study. Given the limited sample size, a parsimonious modeling strategy was adopted, and variables without demonstrated relevance, such as time from symptom onset, were not included in the multivariable model. Blood samples were obtained upon presentation but not standardized by time of day or fasting state, which may influence inflammatory indices. OCT grading was focused on structural findings without correlating with long-term visual outcomes. Furthermore, strict exclusion criteria may reduce applicability to broader populations. Future larger, adequately powered studies are needed to validate these associations and assess their prognostic utility.

## 4. Materials and Methods

### 4.1. Study Design and Setting

We conducted a retrospective observational study at the “Sf. Spiridon” Clinical Emergency County Hospital, Iași, Romania. The study population represented a predominantly Caucasian Eastern European population from Romania. Medical records of patients diagnosed with non-arteritic central retinal artery occlusion between 1 January 2016 and 31 December 2025 were reviewed.

The study was approved by the Research Ethics Committee of “Grigore T. Popa” University of Medicine and Pharmacy, Iași (approval No. 482/27.10.2024), and was conducted in accordance with the Declaration of Helsinki. Given the retrospective design, the requirement for informed consent was waived.

**Figure 1:**
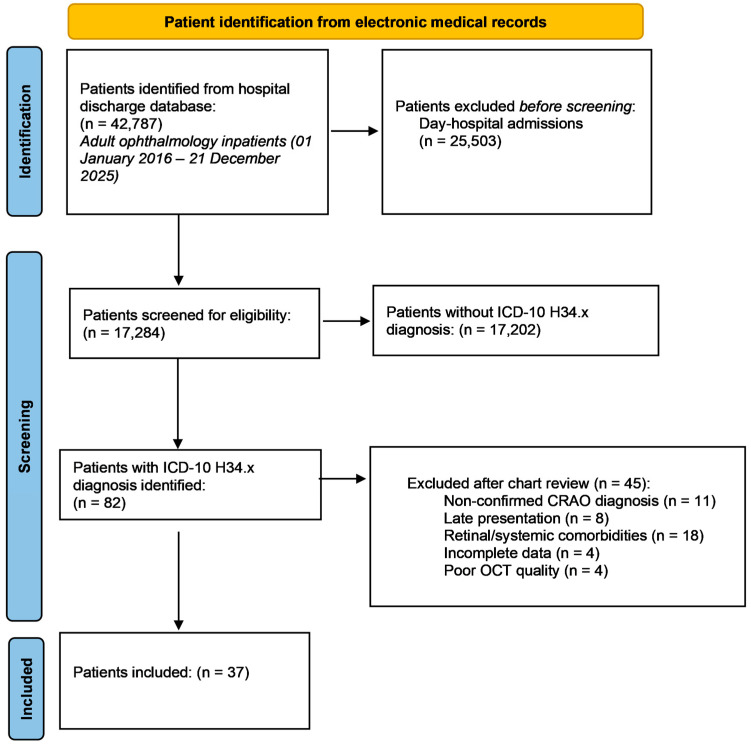
Flow diagram of patient selection.

Consecutive patients were identified from the hospital electronic medical records database using the ICD-10 code group H34.x (retinal vascular occlusions). All retrieved records underwent individual chart review, and only cases with a confirmed diagnosis of NA-CRAO were included based on clinical documentation and available imaging. Figure 1 illustrates the flow diagram of patient selection and exclusion.

Patients were eligible for inclusion if they fulfilled all of the following criteria:

1. Confirmed NA-CRAO based on clinical documentation and available imaging;
2. Acute presentation, defined as ≤168 h from symptom onset;
3. Swept-source OCT at presentation (DRI OCT Triton, Topcon) suitable for OCT severity grading and CMT (Center Thickness) extraction;
4. Complete blood count at presentation that allowed for calculation of inflammatory indices.

Patients were excluded if any of the following were present:

1. Other types of retinal arterial occlusion (branch, isolated cilioretinal, mixed or atypical forms) or arteritic CRAO;
2. Retinal disease, uveitis, glaucoma, ocular trauma, previous ocular surgery, or media opacities precluding reliable OCT acquisition;
3. Conditions or treatments with potential to confound inflammatory indices, including acute infection, malignancy, recent major surgery or trauma (<3 months), major cardiovascular or cerebrovascular disease, advanced renal or hepatic failure, diabetes mellitus, hematologic disorders, autoimmune disorders, current systemic corticosteroid or immunosuppressive therapy or current regular NSAID use;
4. Missing essential clinical, imaging, or laboratory data.

A total of 37 patients met the inclusion criteria and were included in the final analysis.

### 4.2. Data Collection

Demographic variables (age and sex), cardiovascular risk factors (hypertension, dyslipidemia, and smoking status), ophthalmic evaluation at presentation (affected eye, visual acuity, and intraocular pressure), OCT parameters, and laboratory results were retrospectively extracted from electronic and paper medical records using a standardized data collection form. In addition, conventional inflammatory markers, including C-reactive protein and erythrocyte sedimentation rate, routinely assessed at presentation, were collected as part of the standard diagnostic work-up to exclude arteritic CRAO. All data were anonymized prior to analysis.

Best-corrected visual acuity (BCVA) was recorded in Snellen and subsequently converted to logMAR for analysis and reporting, assigning predefined values for low vision (CF 2.10, HM 2.40, LP 2.7) [35].

### 4.3. Optical Coherence Tomography Assessment

Macular OCT performed at presentation using a swept-source device (DRI OCT Triton, Topcon, Tokyo, Japan) was retrospectively analyzed in all included cases. A standardized 3D macular scan (7 × 7 mm, 512 × 256) was used for morphological assessment and OCT severity grading. All scans were reviewed for image quality, centration, and segmentation reliability prior to grading and thickness extraction.

**Figure 2:**
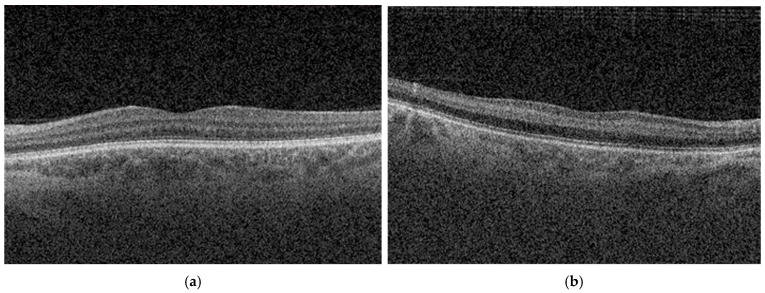

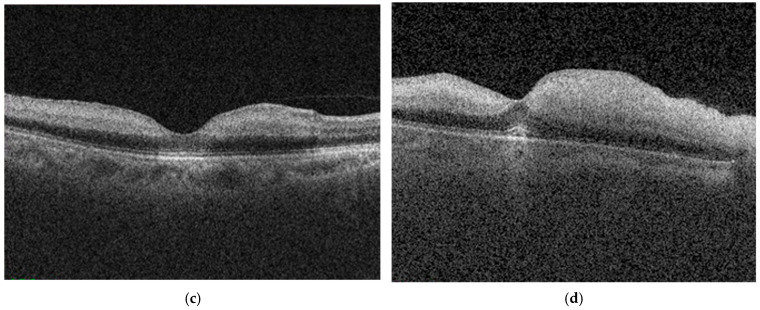
Representative OCT images showing normal macular morphology and OCT-defined ischemic severity in acute NA-CRAO. (**a**) Normal retinal architecture. (**b**) Mild: inner retinal hyperreflectivity without inner retinal thickening, with preserved stratification. (**c**) Moderate: inner retinal hyperreflectivity with inner retinal thickening, with preserved stratification. (**d**) Severe: inner retinal hyperreflectivity with inner retinal thickening and loss of inner retinal layer stratification.

Based on morphological features observed on OCT images at presentation, patients were classified into three severity grades (mild, moderate, and severe) according to the OCT-based classification proposed by Mangla et al. [12]. Mild NA-CRAO was defined as inner retinal hyperreflectivity without inner retinal thickening and with preserved inner retinal layer stratification. Moderate NA-CRAO was defined as inner retinal hyperreflectivity with inner retinal thickening while maintaining preserved stratification. Severe NA-CRAO was defined as inner retinal hyperreflectivity with inner retinal thickening accompanied by loss of inner retinal layer stratification. These grading criteria are summarized in Table 6, and representative OCT B-scans illustrating each severity category are presented in Figure 2.

OCT severity was graded independently by two investigators, with disagreements resolved by consensus. Interobserver reliability for OCT severity classification was evaluated using weighted Cohen’s kappa, which indicates excellent reproducibility (κ = 0.808).

### 4.4. Inflammatory Indices

Complete blood count parameters obtained at presentation, including total leukocyte, neutrophil, lymphocyte, monocyte, and platelet counts, were retrieved from laboratory records. Inflammatory indices were derived from these parameters as follows: the neutrophil-to-lymphocyte ratio was calculated as the ratio between neutrophil and lymphocyte counts, the platelet-to-lymphocyte ratio as platelet count divided by lymphocyte count, and the monocyte-to-lymphocyte ratio as monocyte count divided by lymphocyte count. The systemic immune-inflammation index was defined as the product of platelet and neutrophil counts divided by lymphocyte count, whereas the systemic inflammation response index was calculated as the product of neutrophil and monocyte counts divided by lymphocyte count.

### 4.5. Statistical Analysis

Statistical analyses were performed using SPSS software (version 29.0). Continuous variables were summarized as mean ± standard deviation (SD) or median with interquartile range (IQR) according to data distribution, while categorical variables are presented as counts and percentages Normality of continuous variables was evaluated using the Kolmogorov–Smirnov test. For comparisons between ischemic severity groups, one-way analysis of variance (ANOVA) was applied for normally distributed variables. Homogeneity of variances was assessed using Levene’s test. In case of variance homogeneity, post hoc analysis was performed using the Tukey test; when variances were unequal, the Games–Howell test was applied. For variables with non-normal distributions, the Kruskal–Wallis test was used, followed by pairwise post hoc comparisons with appropriate adjustment for multiple testing.

Associations between inflammatory indices and OCT-based ischemic severity were further evaluated using ordinal logistic regression models. Variables with *p* < 0.10 in univariable analyses were entered into the multivariable model. Effect estimates were reported as odds ratios (ORs) with 95% confidence intervals (CIs). Model performance was assessed using the Nagelkerke R^2^ statistic, and the proportional odds assumption was verified using the test of parallel lines.

A two-tailed *p*-value < 0.05 was considered statistically significant, while *p*-values between 0.05 and 0.10 were interpreted as indicating a statistical trend.

## 5. Conclusions

Systemic inflammatory burden, particularly reflected by NLR and SII, is significantly associated with OCT-defined ischemic severity in acute non-arteritic CRAO. NLR emerged as the dominant systemic predictor, while acute CMT provided complementary structural information on tissue-level injury. These findings highlight the combined role of systemic inflammation and local retinal response in shaping ischemic severity and support the potential utility of integrating inflammatory indices with OCT for early severity stratification.

## Figures and Tables

**Table 1:** Baseline demographic and clinical characteristics according to OCT-based ischemic severity.

Variable	Total (*n* = 37)	Mild (*n* = 7)	Moderate (*n* = 16)	Severe (*n* = 14)	*p*-Value
Age (years, mean ± SD)	65.9 ± 10.5	62.9 ± 13.8	67.8 ± 8.4	65.4 ± 11.2	0.573 ^a^
Male sex, *n* (%)	24 (64.9)	4 (57.1)	12 (75.0)	8 (57.1)	0.530 ^b^
Right eye, *n* (%)	24 (64.9)	5 (71.4)	12 (75.0)	7 (50.0)	0.331 ^b^
IOP (mmHg), median (IQR)	14.0 (12.0–16.5)	13.0 (12.0–17.0)	13.5 (10.0–15.5)	14.0 (12.0–17.3)	0.666 ^c^
BCVA, logMAR, median (IQR)	2.30 (2.00–2.30)	2.30 (0.70–2.70)	2.30 (2.00–2.30)	2.30 (2.00–2.70)	0.564 ^c^
Time from onset (h), median (IQR)	12 (8–33)	10 (8–48)	24 (10–28)	18 (8–39)	0.903 ^c^
CRP (mg/dL), median (IQR)	0.28 (0.188–0.48)	0.22 (0.16–0.30)	0.29 (0.13–0.49)	0.42 (0.22–0.48)	0.353 ^c^
ESR (mm/h), median (IQR)	8.00 (6.50–10.00)	10.00 (4.00–10.00)	8.00 (6.25–9.00)	10.00 (6.75–10.25)	0.309 ^c^
Hypertension, *n* (%)	31 (83.8)	6 (85.7)	14 (87.5)	11 (78.6)	0.794 ^b^
Dyslipidemia, *n* (%)	19 (51.4)	1 (14.3)	11 (68.8)	7 (50.0)	0.055 ^b^
Smoking, *n* (%)	8 (21.6)	0 (0.0)	3 (18.8)	5 (35.7)	0.161 ^b^
Cilioretinal artery present, *n* (%)	6 (16.2)	1 (14.3)	5 (31.3)	0 (0.0)	0.068 ^c^

*p*-values were calculated using the ^a^ one-way ANOVA, ^b^ Pearson’s chi-square test, ^c^ Kruskal–Wallis test. A *p*-value <0.05 was considered statistically significant. IOP = intraocular pressure; IQR = interquartile range; BCVA = best-corrected visual acuity; CRP = C-reactive protein; ESR = erythrocyte sedimentation rate.

**Table 2:** CMT across OCT-based ischemic severity.

Parameter	Mild (*n* = 7)	Moderate (*n* = 16)	Severe (*n* = 14)	*p*-Value
CMT (µm), median (IQR)	254 (247–263)	283 (256–301)	404 (341–481)	<0.001 ^c^

*p*-values were calculated using the ^c^ Kruskal–Wallis test; CMT = central macular thickness; OCT = optical coherence tomography; IQR = interquartile range.

**Table 3:** Systemic inflammatory markers across OCT-based ischemic severity.

Variable	Mild (*n* = 7)	Moderate (*n* = 16)	Severe (*n* = 14)	*p*-Value
WBC (cells × 10^9^/L, mean ± SD)	7.09 ± 2.04	7.78 ± 1.90	8.97 ± 1.60	0.067 ^a^
Neutrophils (cells × 10^9^/L, mean ± SD)	4.10 ± 1.28	4.93 ± 1.39	6.04 ± 1.47	0.013 ^a^
Monocytes (cells × 10^9^/L, mean ± SD)	0.60 ± 0.24	0.68 ± 0.09	0.64 ± 0.22	0.576 ^a^
Lymphocytes (cells × 10^9^/L, mean ± SD)	2.19 ± 0.70	1.94 ± 0.61	2.14 ± 0.56	0.550 ^a^
Platelets (cells × 10^9^/L, mean ± SD)	252 ± 64	231 ± 32	264 ± 70	0.258 ^a^
NLR (mean ± SD)	1.89 ± 0.42	2.69 ± 0.80	3.02 ± 1.08	0.029 ^a^
MLR (mean ± SD)	0.29 ± 0.11	0.39 ± 0.14	0.31 ± 0.11	0.122 ^a^
PLR (mean ± SD)	125.9 ± 45.3	132.6 ± 52.2	127.8 ± 31.6	0.929 ^a^
SII (mean ± SD)	480.6 ± 169.0	621.2 ± 204.5	773.8 ± 248.9	0.018 ^a^
SIRI (median, IQR)	857.6 (610.6–1150.4)	655.8 (397.3–912.7)	705.0 (610.6–1167.4)	0.101 ^c^

*p*-values were calculated using the ^a^ one-way ANOVA, ^c^ Kruskal–Wallis test. NLR = neutrophil-to-lymphocyte ratio; MLR = monocyte-to-lymphocyte ratio; PLR = platelet-to-lymphocyte ratio; SII = systemic immune-inflammation index; SIRI = systemic inflammation response index; IQR = interquartile range.

**Table 4:** Univariable ordinal logistic regression analyses for OCT-based ischemic severity.

Variable	OR	95% CI	*p*-Value
CMT	1.040	1.018–1.063	<0.001
WBC (×10^9^/L)	1.493	1.051–2.119	0.025
Neutrophil count (×10^9^/L)	1.948	1.224–3.099	0.005
NLR	2.659	1.240–5.703	0.012
SII	1.004	1.001–1.008	0.005
Dyslipidemia	0.567	0.167–1.916	0.361
Presence of cilioretinal artery	2.852	0.537–15.120	0.218

OR = odds ratio; CI = confidence interval; CMT = central macular thickness; NLR = neutrophil-to-lymphocyte ratio; WBC = white blood cell count; SII = systemic immune-inflammation index.

**Table 5:** Multivariable ordinal logistic regression. CMT = central macular thickness; NLR = neutrophil-to-lymphocyte ratio.

Variable	OR	95% CI	*p*-Value
CMT	1.052	1.022–1.083	0.001
NLR	8.398	1.799–39.213	0.007

Higher NLR values were associated with greater odds of severe ischemia (OR: 8.398; 95% CI: 1.799–39.213; *p* = 0.007). CMT was also significantly related to increasing ischemic severity (OR: 1.052; 95% CI: 1.022–1.083; *p* = 0.001). The model showed strong explanatory performance (Nagelkerke R^2^ = 0.804) and fulfilled the proportional odds assumption (test of parallel lines, *p* > 0.05).

**Table 6:** OCT-based severity classification of acute NA-CRAO.

OCT Characteristic	Mild CRAO	Moderate CRAO	Severe CRAO
Inner retinal hyperreflectivity	Present	Present	Present
Inner retinal thickening	Absent	Present	Present
Inner retinal layer stratification	Preserved	Preserved	Lost

OCT = optical coherence tomography; NA-CRAO = non-arteritic central retinal artery occlusion.

## Data Availability

The original contributions presented in this study are included in the article. Additional information may be obtained from the corresponding author upon reasonable request.

## Abbreviations

The following abbreviations are used in this manuscript:

ANOVA	Analysis of Variance
BCVA	Best-Corrected Visual Acuity
CBC	Complete Blood Count
CF	Counting Fingers
CI	Confidence Interval
CMT	Central Macular Thickness
CRAO	Central Retinal Artery Occlusion
CRP	C-reactive protein
ESR	Erythrocyte sedimentation rate
HDL-C	High-Density Lipoprotein Cholesterol
HM	Hand Motion
ICD-10	International Classification of Diseases, 10th Revision
IOP	Intraocular Pressure
IQR	Interquartile Range
logMAR	logarithm of the Minimum Angle of Resolution
LP	Light Perception
MLR	Monocyte-to-Lymphocyte Ratio
NA-CRAO	Non-Arteritic Central Retinal Artery Occlusion
NLR	Neutrophil-to-Lymphocyte Ratio
OCT	Optical Coherence Tomography
OR	Odds Ratio
PLR	Platelet-to-Lymphocyte Ratio
RAO	Retinal Artery Occlusion
SD	Standard Deviation
SII	Systemic Immune-Inflammation Index
SIRI	Systemic Inflammation Response Index
WBC	White Blood Cell count
